# Real-Time Detection of Intruders Using an Acoustic Sensor and Internet-of-Things Computing

**DOI:** 10.3390/s23135792

**Published:** 2023-06-21

**Authors:** Najeeb Al-Khalli, Saud Alateeq, Mohammed Almansour, Yousef Alhassoun, Ahmed B. Ibrahim, Saleh A. Alshebeili

**Affiliations:** 1 KACST-TIC in Radio Frequency and Photonics for the e-Society (RFTONICS), King Saud University, Riyadh 11421, Saudi Arabia; 2 King Abdullah Institute for Nanotechnology (KAIN), King Saud University, Riyadh 11451, Saudi Arabia; 3 Electrical Engineering Department, King Saud University, Riyadh 11421, Saudi Arabiaeng.mohammed481@gmail.com (M.A.); yhassoun@ksu.edu.sa (Y.A.)

**Keywords:** intruders detection, real-time implementation, acoustic sensor, Internet of Things, adaptive thresholding

## Abstract

Modern home automation systems include features that enhance security, such as cameras and radars. This paper proposes an innovative home security system that can detect burglars by analyzing acoustic signals and instantly notifying the authorized person(s). The system architecture incorporates the concept of the Internet of Things (IoT), resulting in a network and a user-friendly system. The proposed system uses an adaptive detection algorithm, namely the “short-time-average through long-time-average” algorithm. The proposed algorithm is implemented by an IoT device (Arduino Duo) to detect people’s acoustical activities for the purpose of home/office security. The performance of the proposed system is evaluated using 10 acoustic signals representing actual events and background noise. The acoustic signals were generated by the sounds of keys shaking, the falling of a small object, the shrinking of a plastic bag, speaking, footsteps, etc. The effects of different algorithms’ parameters on the performance of the proposed system have been thoroughly investigated.

## 1. Introduction

The Internet of Things (IoT) is an emerging paradigm that enables communication between electronic devices and sensors. IoT uses smart devices and the internet to provide innovative solutions to various challenges and issues related to various businesses and governmental and and public/private industries across the world [1]. IoT is progressively becoming an important aspect of our life, where an extensive variety of smart systems, frameworks, intelligent devices, and sensors are currently used. Cisco anticipated that by the year 2022 there would be more than 28 billion IoT-connected devices, as compared to 18 billion in 2017, where more than half of those devices would be machine-to-machine connections [2]. It is also anticipated that the number of connected IoT devices will reach 50 billion devices by 2030 [3].

Security has been one of the most critical social challenges in recent years. This is due to a spike in instances of robbery and intrusion in practically every location, including homes and offices. Intruders can cause a considerable loss of belongings and may also commit additional crimes. Therefore, intruder detection systems are of utmost importance and an unavoidable requirement of our daily life.

IoT systems are among the modern home automation systems’ security-enhancing capabilities. These devices can provide a revolutionary home security system that can detect intruders by using an appropriate sensing device and instantly notify the property owner. Several sensors can be utilized to detect intruders in different environments and situations, such as video sensors, passive infrared (PIR) sensors, contact sensors, acoustic sensors, pressure sensors, and vibration sensors. Table 1 shows a number of the sensors used in intruder detection systems. The effectiveness of the selected sensor depends on a variety of factors, including the type of sensor, the placement of the sensor, and the environment in which the sensor is used.

Monitoring cameras are the most common video sensors for intruder detection [4,5]. This traditional system is a viable solution for home and building security but requires several sensors to cover different nearby and separate areas. In addition, visible cameras need light to work, while infrared cameras, which are well suited for dark places, are quite expensive. Furthermore, the use of a camera poses a threat in the sense of violating the privacy of a place, possibly through unauthorized access to the footage of the place where the camera is installed. Further, cameras can also be recognized by an intruder.

Motion sensors are another commonly used sensor in intruder detection systems. These sensors detect movement within a specified area. There are several types of motion sensors, including PIR sensors, ultrasonic sensors, and microwave sensors. PIR sensors are the most widely used motion sensors due to their low cost and high accuracy. However, motion sensors have limitations such as blind spots and range limitations [6,7].

Contact sensors are another commonly used sensors in intruder detection systems to monitor the opening and closing of doors, windows, and other entry points. These sensors are made up of two components, a magnetic piece and a sensor body, which are located separately. They work by detecting changes in magnetic fields when a door or window is opened. Contact sensors are relatively simple and inexpensive, making them a popular choice for home security systems. They are also easy to install and require little maintenance. However, contact sensors do have some limitations. They only detect when a door or window is opened but do not detect motion or other types of intrusion. Pressure sensors, on the other hand, are used to detect changes in pressure or weight and can be used to detect the presence of intruders. These sensors are often placed under carpets or floor mats and trigger an alarm or alert when pressure is applied [7].

Radar is another sensor that can be used to detect intruders. It operates by radiating electromagnetic energy and detecting the echo returned from reflecting objects (targets). The nature of the echo signal provides information about the target. The range, or distance, to the target is found from the time it takes for the radiated energy to travel to the target and come back [8]. Despite all the advantages of radar, it is not recommended for indoor intruder detection because it is an active device causing electromagnetic radiation. Further, it works properly only in the line-of-site transmission and is difficult to hide from intruders. Another intrusion detection system could be based on the scattered reflections generated by a pulsed light or acoustic signals [9,10,11]. This concept is analogous to the radar challenge of transmitting a pulsed signal. The scattered signal reflections are analyzed for the purpose of intruder detection and localization. This solution is o quite high in cost and suitable for outdoor applications. Seismic vibration sensor technology can also be a viable contender for the detection of human footsteps and hence an intruder [12]. The amplitude of propagating waves in the soil caused by the impact of a person’s movement is measured using seismic sensors. A seismic sensor can detect a wide frequency band that is below the threshold of the human hearing spectrum. Geophones are sensors used to measure the amplitude of seismic waves within the soil. This solution is well-suited for outdoor applications. Further, the sensor needs to be immersed in the ground.

Several studies have attempted the problem of intruder detection using acoustic approaches. In [13], the authors used linear frequency modulation (LFM) as an acoustic source signal with the employment of coherence bandwidth for the sake of acoustic-based intruder detection. In [9], they exploited a source of white noise and an array of microphones for the purpose of intruder detection. After white noise is propagated, two selected features out of the source and received signals are compared which are based on the Short-Time Fourier Transform (STFT) and the zero-crossing rates. The presence of an intruder is determined based on the comparison result if it exceeds a certain threshold. Similarly, in [14], the authors compared the energy of a chirp signal and its reverberation based on a two-dimensional spectro-temporal filtering mechanism of the Fourier spectrogram. Thus, they can detect any changes in the acoustic scene affecting the propagated signal. In [15], the authors proposed a method for intruder detection using a speaker and microphone in addition to a camera. Their acoustic detection algorithm is based on measuring the distortion of the space transfer function so that the magnitude of the recorded signal is much lower than the magnitude of the propagated signal in the case of the presence of an intruder. Note that the aforementioned research methods, which are proposed for intruder detection, have the disadvantage of requiring an active acoustic source to work. Second, they lack real-time implementation of their intruder detection systems, which is crucial for these types of applications.

**Table 1:** Sensors used in intruder-detection systems.

Sensor	Reference	Limitation
Video	[4,5]	Requires several sensors to cover different areas that are nearby and apart Needs light to work Infrared camera is quite expensive Poses a threat in the sense of violating the privacy of a place
Motion	[6,7]	Blind spots Range limitations
Magnetic contact	[7]	Do not detect motion or other types of intrusions
Radar	[8]	Not recommended for indoor intruder detection Works properly only in the line-of-site transmission
Pulsed light	[9,11]	Quite high cost Not suitable for indoor applications
Vibration	[12]	Not suitable for indoor applications
Acoustic	[9,13,14,15]	Affected by environmental noise

In this research work, a real-time smart security system based on an IoT device is proposed, which continuously monitors the area and notifies the authorized person(s) in the event of intrusion. A main objective in our development is to come up with a cost-effective, privacy-preserving, and reliable intruder detection system that is well suited for indoor applications. Specifically, the goals are (1) developing a computationally less demanding intruder detection algorithm that can be executed by an IoT device, (2) employing IoT sensors that ensure some privacy for homes and other private places, and (3) employing IoT sensors covering wider areas of nearby but separated places. The intruder detection system proposed in this research work is developed such that it sends a notification to the user’s phone once an intruder is detected over at least 10 consecutive positive decisions for more reliable results. It does so in less than 3 s, which meets the real-time requirement of the application at hand, as demonstrated in Section 3.2.

The remaining parts of this paper are organized as follows. The system concept, detection algorithm employed in this research work, and hardware implementation are described in Section 2. The experimental investigation is discussed and presented in Section 3. Concluding remarks are given in Section 4.

## 2. System Development

### 2.1. System Concept

**Figure 1:**
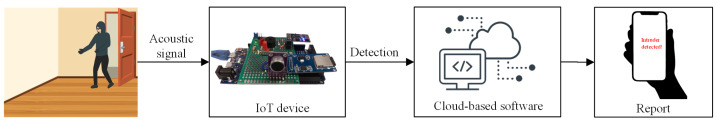
System Concept.

Figure 1 represents the main concept of our solution for the problem at hand. In particular, there is an acoustic sensor that is placed in the area to be protected. The acoustic signal captured by the sensor is digitized and processed in an IoT device. The main function of the IoT device is to detect abnormal activities in the digitized acoustic signal. When no intruder is present, the acoustic signal is pure background noise. This background noise is used in the initialization phase of the IoT device for computing the parameters of the intruder detector. Later, the presence of an intruder will produce an acoustic signal, which can be detected with a proper thresholding process. In this study, we consider the use of an adaptive algorithm for detecting abnormalities in a real-time captured acoustic signal. The adaptive algorithm makes use of an adaptive threshold to detect intruders and is implemented on a dedicated IoT hardware to speed up the computational process. One option for such a hardware is the Arduino Due chip, which is an integrated circuit designed to have the flexibility to be programmable based on the algorithm to be implemented [16]. Once an intruder detection is declared, or equivalently the energy of the acoustic signal crosses the adaptive threshold, a wireless control module (ESP8266) is used with the Arduino Due for sending a notification to the intended person’s mobile, through the Blynk IoT cloud, as demonstrated in Figure 1.

### 2.2. Detection Algorithm

Intruder detection using acoustic sensors can be formulated as a classical binary testing problem. The detector usually stores a threshold to determine if the acoustic signal is high enough to be caused by the presence of an intruder. A decision is made in favor of the intruder state if the signal value exceeds the threshold. Otherwise, it is set in favor of the no-intruder state. Note that a threshold of a fixed value may cause an excessive number of false alarms. This is because noise alone might exceed low-level thresholds, leading in such a case to a false alarm. In contrast, if the threshold is set too high, weak intruder signals might not be detected; this latter situation is classified as miss detection. For reliable intruder detection in a noisy environment, the threshold must be varied adaptively according to the background noise.

**Figure 2:**
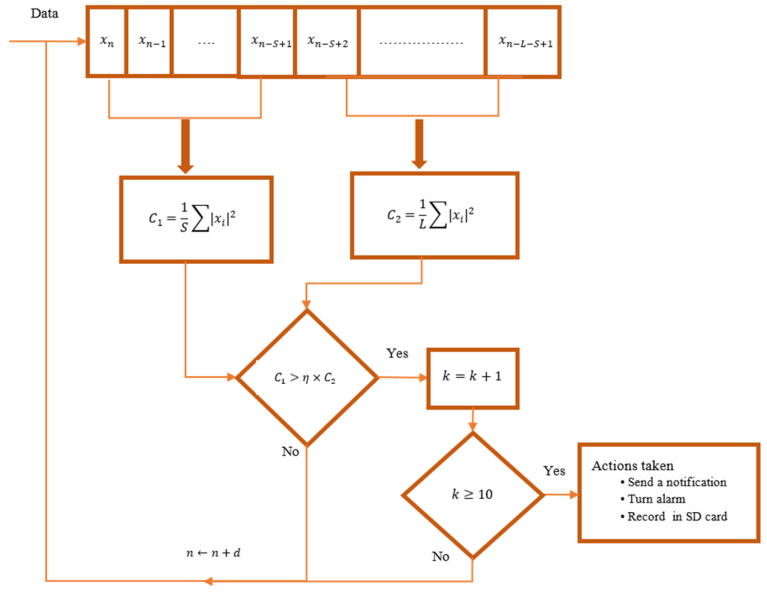
The Architecture of the STA/LTA Algorithm.

In this study, we propose a detection method using the “short-time-average through long-time-average” (STA/LTA) algorithm [17]. This algorithm computes the threshold in an adaptive manner to maintain a constant false alarm rate (CFAR). It continuously calculates the average value of the energy of an acoustic signal in two consecutive time-moving windows. The short-time-average window (STA) estimates the acoustic events, while the long-time-average window (LTA) provides an estimation of the temporal energy of the acoustic background noise of the surrounding environment. The algorithm works by comparing the value of the STA window to the value of the LTA window multiplied by a scaling constant based on the desired probability of a false alarm. When the value of an STA window exceeds that of the LTA window, an event is “declared”. The equation governing the operation of STA/LTA algorithm is given by [18]:

(1) $$\frac{1}{S}\sum_{i=n-S+1}^{n}{\left|X_{i}\right|}^{2}≷\frac{\eta }{L}\sum_{i=n-L-S+1}^{n-S}{\left|X_{i}\right|}^{2}$$

where the right-hand term of Equation (1) is the adaptive threshold, $x_{i}$ is the *i*th sample of digitized acoustic signal, *S* is the length of STA window, *L* is the length of LTA window, and η represents a predefined scaling factor. The symbol ≷ means that an intruder is present if the value of the left-hand side of Equation (1) is greater than that of the right-hand side. If it is less, then a no-intruder state is declared. For proper operation of the STA/LTA algorithm in a particular application, *S*, *L*, and η must be carefully selected. The size of the LTA window is often kept to 5 to 10 times the size of STA window [18]. The STA window is usually selected depending on the short event duration. Unfortunately, it is not possible to determine the type of such an event in advance as it could be a human voice, a sound originating from opening doors, and/or a sound originating from breaking items. Therefore, the size of the STA window is set heuristically. On the other hand, the value of the predefined scaling factor η is adjusted so that it maintains a pre-defined constant false alarm rate. In particular, we run the STA/LTA algorithm with a selected window size over background signals recorded at the place to be monitored. The value of η is varied until the desired constant false alarm rate is reached. Figure 2 shows the architecture of STA/LTA, where *d* is the step size and *k* is the number of consecutive positive decisions employed to reduce the number of false events. In particular, the algorithm considers the presence of an intruder if *k* consecutive positive decisions are made. A warning message is then released by the system based on the user’s requirements.

### 2.3. Hardware Implementation

**Figure 3:**
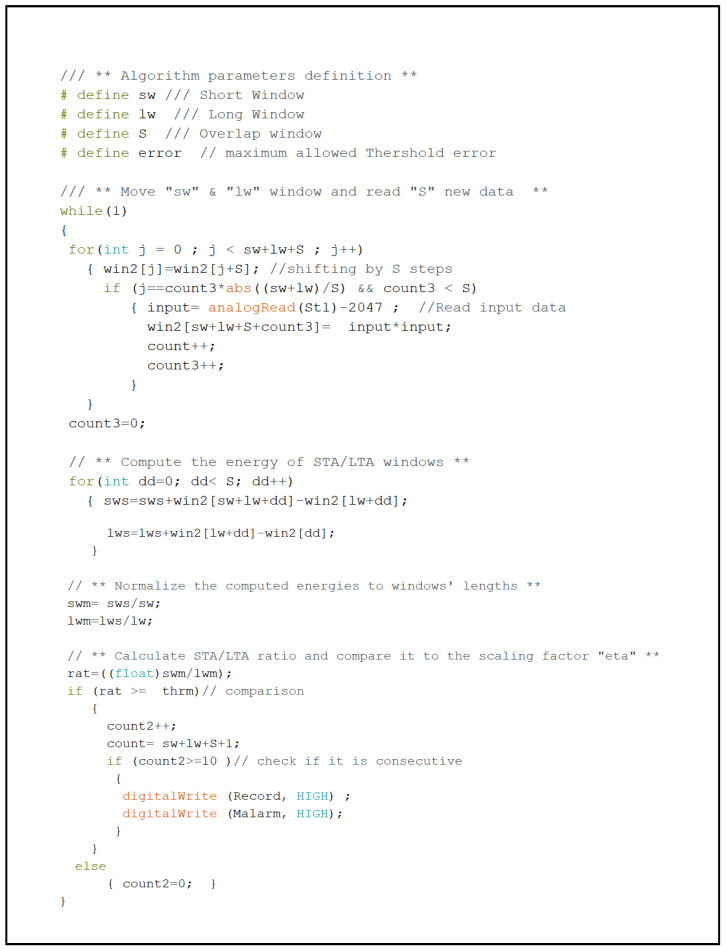
Arduino code.

Implementing the STA/LTA algorithm in a small controller to detect intruders using acoustic signals implies dealing with real-time data streaming that must be processed within a given time constraint, called real-time stream processing. This means that the system should be able to do the following tasks in real time: collecting the acoustic signal from ADC and storing it in a particular place of the STA/LTA windows arrays, shifting the STA/LTA windows arrays, summing the two new windows’ arrays, averaging, calculating the ratio, and comparing it with a predefined threshold. Among the mentioned tasks, reading new data and shifting and summation of windows represent the bottlenecks in realizing a real-time streaming process, especially for the microcontrollers that have a single core and can only execute one instruction at a time. Therefore, an Arduino code was developed in a way that switches between the operations of shifting windows and reading new data within an acceptable rate, whereas the summation operation of the windows was reduced to add and subtract *d* samples that entered and exited each window. The code of the STA/LTA algorithm is as shown in Figure 3.

**Figure 4:**
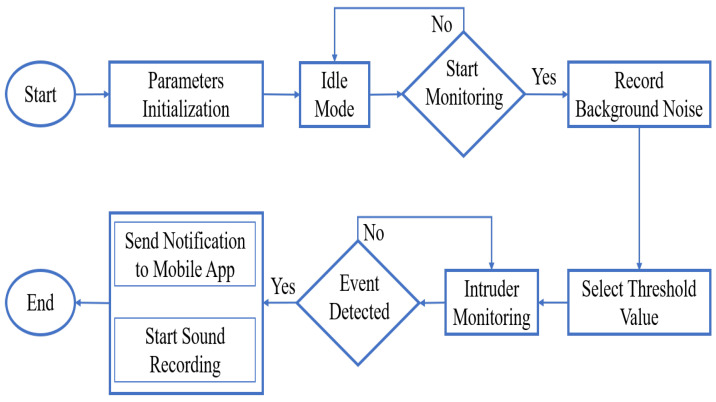
Intruder detection flowchart.

Figure 4 shows the intruder-detection workflow. The system starts by initializing the hardware and uploading the predefined algorithm’s parameters. The values of these parameters were specified using an offline simulation study, the details of which are given in the next section. Then, the system goes into idle mode until a turn-on signal was received from the mobile application. Once the turn-on signal is received by the system, the monitoring process is started; firstly, the background acoustic noise is recorded on the SD card for a specified time and used afterward by the system to select the suitable scaling factor η according to the pre-specified constant false alarm rate. Then, the input acoustic signal is continuously captured and stored on the STA/LTA window array while the shifting process is performed during this interval of time. After that, the summation and averaging processes are performed, and the ratio of the values of STA and LTA windows is compared with the scaling factor η. If the ratio exceeds the value of the scaling factor, the event is declared, and the alarm signal is sent to the intended person’s mobile to alert them about the presence of an intruder. Finally, when there is a presence of an intruder, the system starts recording the acoustic signal on the SD card for a certain time defined by the user.

**Figure 5:**
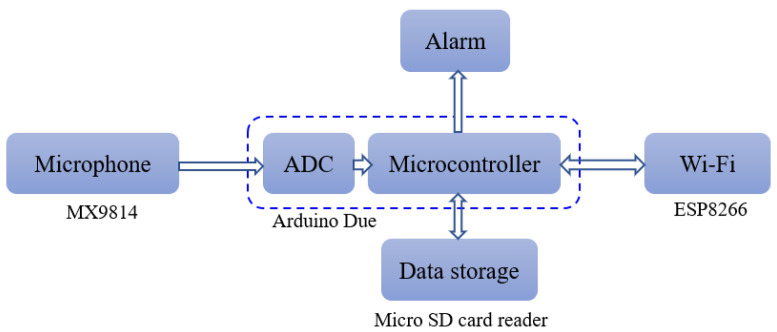
System hardware block diagram.

**Figure 6:**
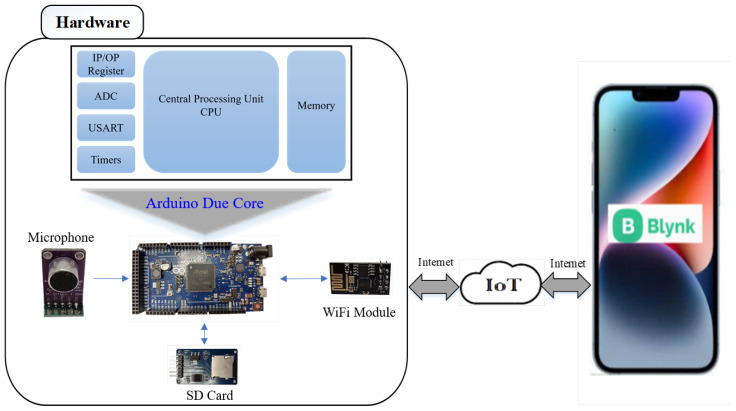
System hardware architecture.

**Figure 7:**
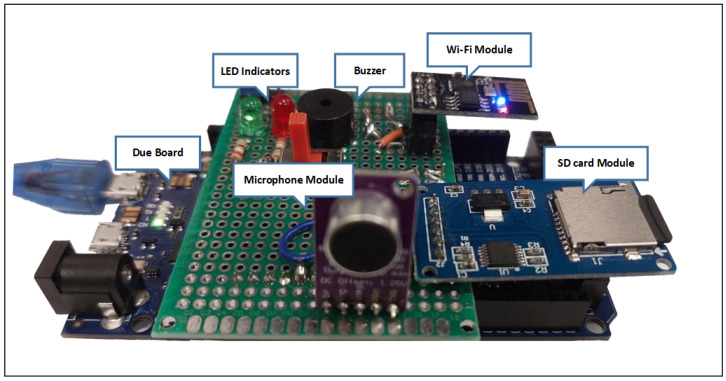
Intruder’s detector prototype.

This advanced electronics technology offers a wide spectrum of microcontrollers that are available off the shelf. Among them, we chose the Arduino platform. The Arduino is an open-source computer hardware/software platform for building digital devices and interactive objects that can sense and control the physical world around them. The Arduino device consists of a microcontroller (usually an Atmel AVR or ARM processor), a set of digital and analog input/output pins, and a development environment that includes an integrated development environment (IDE) and a library of pre-written software functions that can be used to control the board’s various inputs and outputs. Many versions of the official Arduino hardware have been commercially produced to date. Arduino Due has the best features that allow an efficient implementation of the STA/LTA algorithm. With Arduino Due, the system can achieve an average processing time as low as 47.6 μs with a window size of 10,000 samples (STA window + LTA window). Figure 5 shows the system hardware block diagram. A MAX9814 microphone amplifier module is used as an acoustic sensor with sensitivity in the range of −44 dBV/Pa to −26 dBV/Pa and a frequency response range of 20 Hz to 20 kHz. This module is a low-cost, high-quality microphone amplifier with automatic gain control (AGC) and low-noise microphone bias [19]. A wireless control module (ESP8266) is used with Arduino Due. This module uses a Wi-Fi channel, which is integrated into the IoT system. A notification alarm is sent via a Wi-Fi network to the Blynk IoT cloud and then collected by an intended person’s mobile through a cloud-based mobile application. Finally, an SD card adapter module is used to record the acoustic signals. This module has a serial peripheral interface (SPI) to connect with the microcontroller. Figure 6 shows the intruder detection system hardware architecture. In the beginning, we wrote a code using the Arduino IDE, which controls the communication between the hardware components. This code is then compiled and uploaded to the hardware board, where it runs the various hardware components. In particular, the Arduino first receives the acoustic signal captured by the MAX9814 module and converts it to a digital signal using the Arduino build-in ADC. After that, the received signal is either stored in the SD card or analyzed using the implemented adaptive algorithm, as shown in Figure 5. Depending on the algorithm’s calculation outputs, the Arduino sends a notification signal to the mobile application if an intruder is detected, using the WiFi module (ESP8266) and the IoT cloud. Figure 7 shows the overall intruder’s detection system prototype.

## 3. Experimental Results

### 3.1. Selection of Algorithm’s Parameters

In this subsection, we describe experiments we performed to select the parameters of the STA/LTA algorithm for best performance using real acoustic data recorded by Arduino Due. We have considered the probability of detection and probability of miss for the performance validation at the fixed probability of a false alarm. The probability of detection is an important parameter in many fields, which refers to the likelihood of correctly identifying the presence of a target signal or event. The probability of a miss, on the other hand, is also an important metric, particularly in the areas where the consequences of a missed detection can be significant. It is a measure of the likelihood that a particular event or object will not be detected or identified by a given system. In our development, the STA/LTA is adjusted to have a pre-defined constant false alarm rate, a performance metric used to evaluate the likelihood of a system flagging an event as occurring when this is not true.

Next, acoustic background noise and ten different events were recorded for 3 min and stored on the SD card. The system recording sampling rate was 14 KHz with a resolution of 12 bits. These records were analyzed using MATLAB codes to select the proper main parameters for the STA/LTA algorithm.

**Figure 8:**
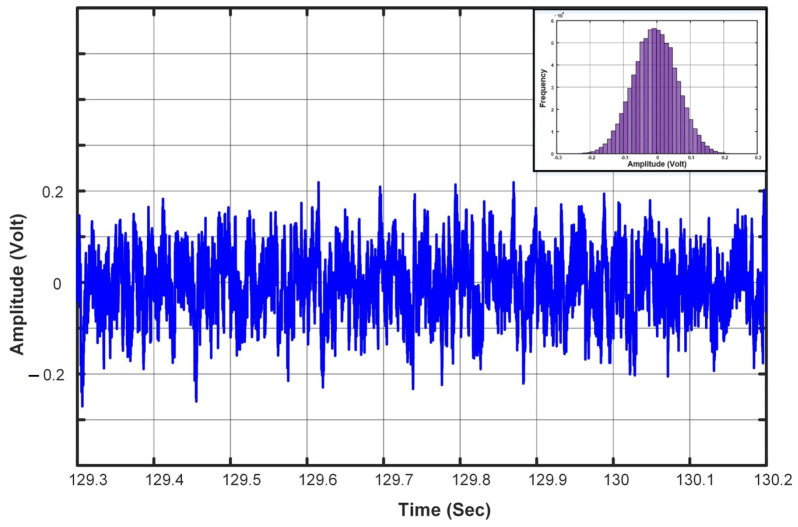
Segment of a background acoustic signal with its histogram.

The background acoustic noise was recorded at a laboratory when no individuals were available. Figure 8 shows a snapshot of the background. As can be seen from the figure, the background noise closely follows the Gaussian distribution, as shown in the inset of Figure 8.

**Figure 9:**
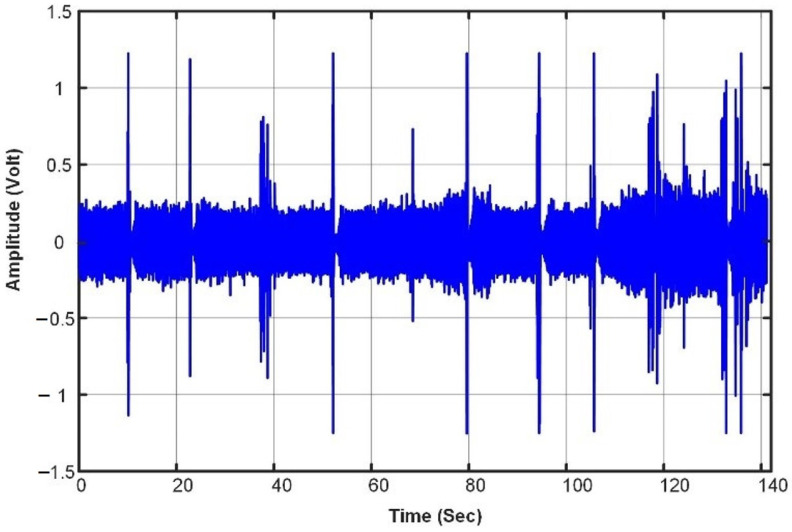
Acoustic signal with 10 events.

**Figure 10:**
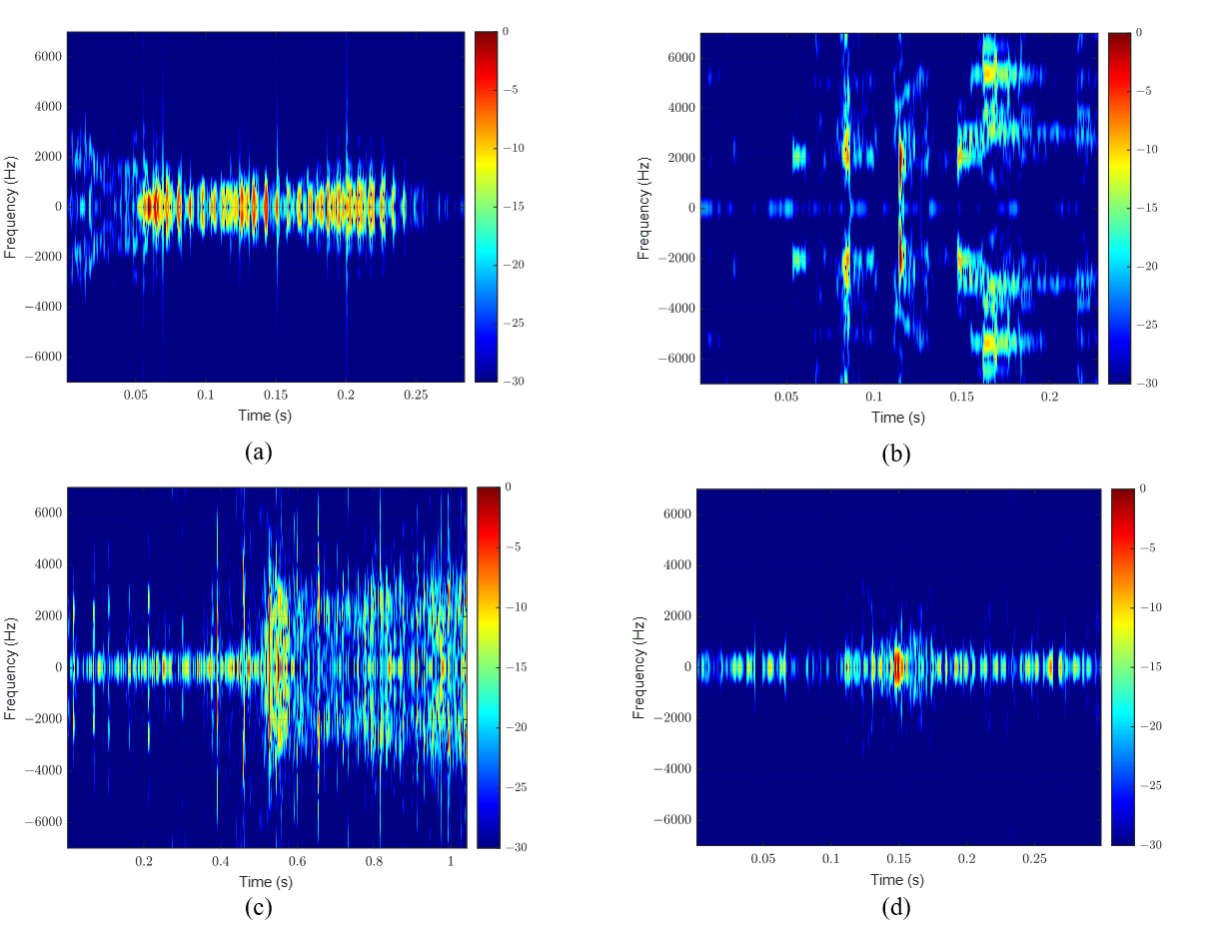
Spectrograms of four events: (**a**) speaking, (**b**) keys, (**c**) plastic shrinking, (**d**) footsteps.

Ten events were recorded at the same laboratory, where each event was repeated ten times with a gap of almost ten seconds between consequence events. Table 2 shows the recorded events’ details and the average duration of each. As can be seen from the table, the maximum event duration is 1.036 s for the acoustic signal generated by the plastic bag, the minimum duration is 0.043 s for the acoustic signal generated by the light switch, and the average calculated duration of all events is 0.49 s. Figure 9 shows an acoustic signal that contains the ten different events that were extracted from the originally recorded signals. These recorded data are used with the developed MATLAB codes to evaluate the performance of the STA/LTA algorithm and to select the proper algorithm’s parameters that will be implemented in the system hardware. Figure 10 further shows the spectrograms of four events with different durations.

The detection performance of the proposed system was evaluated using recorded acoustic signals representing the actual events and background noise. The effects of different algorithm parameters on the detection performance of the proposed system have been thoroughly investigated. The evaluation was based on the following three metrics: the probability of detection, the probability of misdetection, and the probability of false alarm.

**Figure 11:**
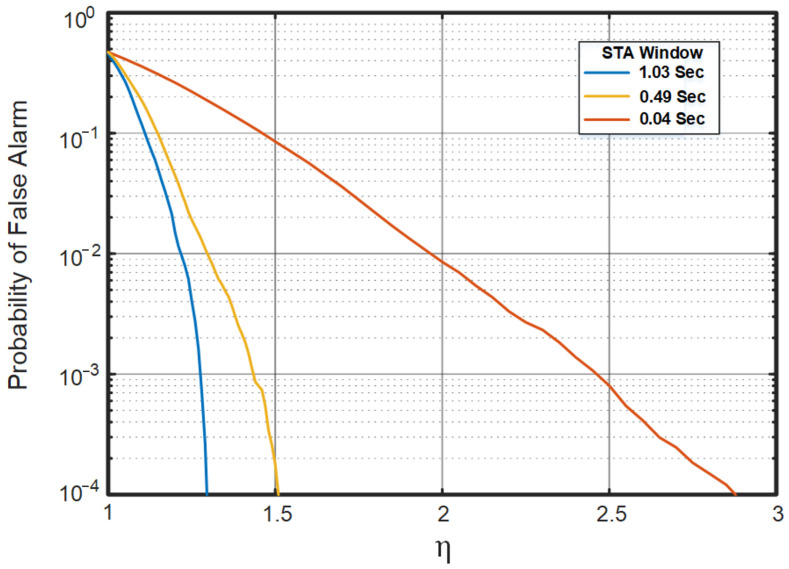
Probability of a false alarm versus the scaling factor η.

In this study, three different STA window sizes were used to evaluate the performance of the algorithm. These windows were selected depending on the minimum, average, and maximum duration of the previously recorded events. Initially, the LTA window was kept at 5 times the selected STA window, and the moving window step size was held at *d* = 14 samples, which is equivalent to 0.001 of the sampling rate. However, the scaling factor η was chosen to have a 0.001 probability of a false alarm. The values of η were determined by running the STA/LTA algorithm over the recorded background signal. Figure 11 shows the plot of the probability of a false alarm against the change in the value of η for the three different STA window sizes. The values of η for the minimum, maximum, and average STA window sizes are 2.47, 1.28, and 1.44, respectively.

**Figure 12:**
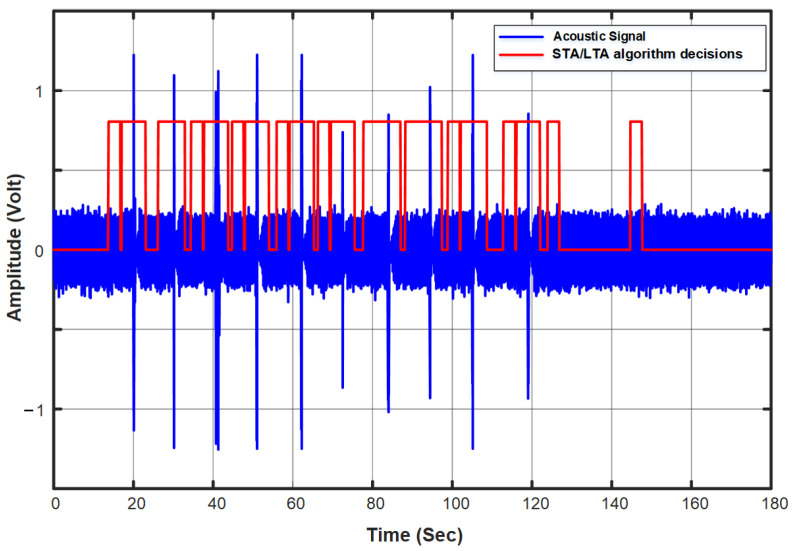
Keys’ acoustic signal with the STA/LTA algorithm decisions.

The detection performance was computed from the ten recorded acoustic signals, where we found that all events in the ten different events were perfectly detected with no miss using the three predefined STA window sizes. Table 3 shows the details of the results. However, the algorithm showed quite a high number of false alarms depending on the STA window size used. Figure 12 shows the keys’ acoustic signal with the STA/LTA algorithm decisions displayed in red over the acoustic signal.

These false alarms can be reduced by either increasing the value of η or considering the presence of abnormality if the detection ratio exceeds the threshold, for example, in *k* consecutive runs. The performance of the algorithm is re-evaluated using four *k* values (1, 5, 10, and 20). The STA/LTA algorithm results for the different values of *k* are shown in Table 4. Improvement in performance is pronounced only when the STA window was 0.043 s. Table 4 shows the performance when the signal generated by the keys is considered. It is observed that the number of false events decreases as the value of *k* increases. However, selecting the proper value of *k* is required to avoid missing real events, especially those of short duration. In our study, we found that *k* = 10 is a suitable value to reduce the false alarm without missing real events of the recorded acoustic signals.

Finally, we varied the value of step size *d* to determine its effect on the algorithm performance. The studied step sizes are 14, 28, 42, and 56 samples, which represent 0.001, 0.002, 0.003, and 0.004 of the recording sampling rates, respectively. It can be seen from Table 5 that increasing the step size results in reducing the number of false events. Furthermore, increasing the step size *d* beyond a certain value will result in missing some events. Therefore, the step size *d* must be selected carefully. It must be mentioned here that selecting the step size is affected by the value of *k* value, where the multiplication of these two values must not be smaller than the expected duration of the shortest event.

### 3.2. Hardware Demonstration

The performance of the developed system was evaluated extensively in real environments using an Arduino IDE serial monitor and MATLAB-developed code. The evaluation shows the calculation of the system’s scaling factor η and presents results for intruder detection as well as the system speed in a real-time environment. The system was tested at a laboratory when no individuals were available to compute the proper value of η and when an event occurs to compute the detection performance.

Two scenarios have been considered to test the developed prototype. The first scenario tests the detection system prototype by comparing the results of each of its main functions with those produced by the MATLAB code. The second scenario tests all the functions of the system prototype together.

In the first scenario, five background noise records of 100 s length were stored in the SD card. This duration is sufficient to estimate the values of η for a probability of a false alarm of value 0.001. Table 6 shows the estimated scaling factor η and the average calculation time per sample for each of the five recorded background noises when threshold computations were performed by the prototype itself and by the MATLAB code. It can be seen from the table that the values of η for both methods are identical, which validates the background calculation using the prototype. It is intuitively not surprising to note that the speed of MATLAB’s computation of η is far higher than that of the prototype because MATLAB utilizes an Intel Core i5 processor of a laptop with 2.67 GHz speed and 8 GB memory, while the prototype utilizes Arduino Duo, a simple controller with very limited capabilities.

For event detection, five records each of three minutes were stored on the SD card. Each record contains five events selected from the ten events given in Table 2. Table 7 shows the number of detected events using both MATLAB and the prototype. Both methods perfectly detect the presence of an event.

The second scenario addresses the real-time evaluation of the whole prototype. In this scenario, the lengths of STA and LTA windows were set to 0.043 and 0.215 s, respectively, and the step size (*d*) of moving window was set to 0.003 of the sampling rate. Two steps were executed:

1. The prototype was set to record 100 s of background noise, and then the STA/LTA algorithm was run over the recorded background signal to compute the threshold η.
2. A total of 20 different events (selected from the events mentioned in Table 2) were generated inside the lab and within a distance of 1, 2, and 5 m from the prototype location. All generated events were captured and processed by the proposed prototype.

**Figure 13:**
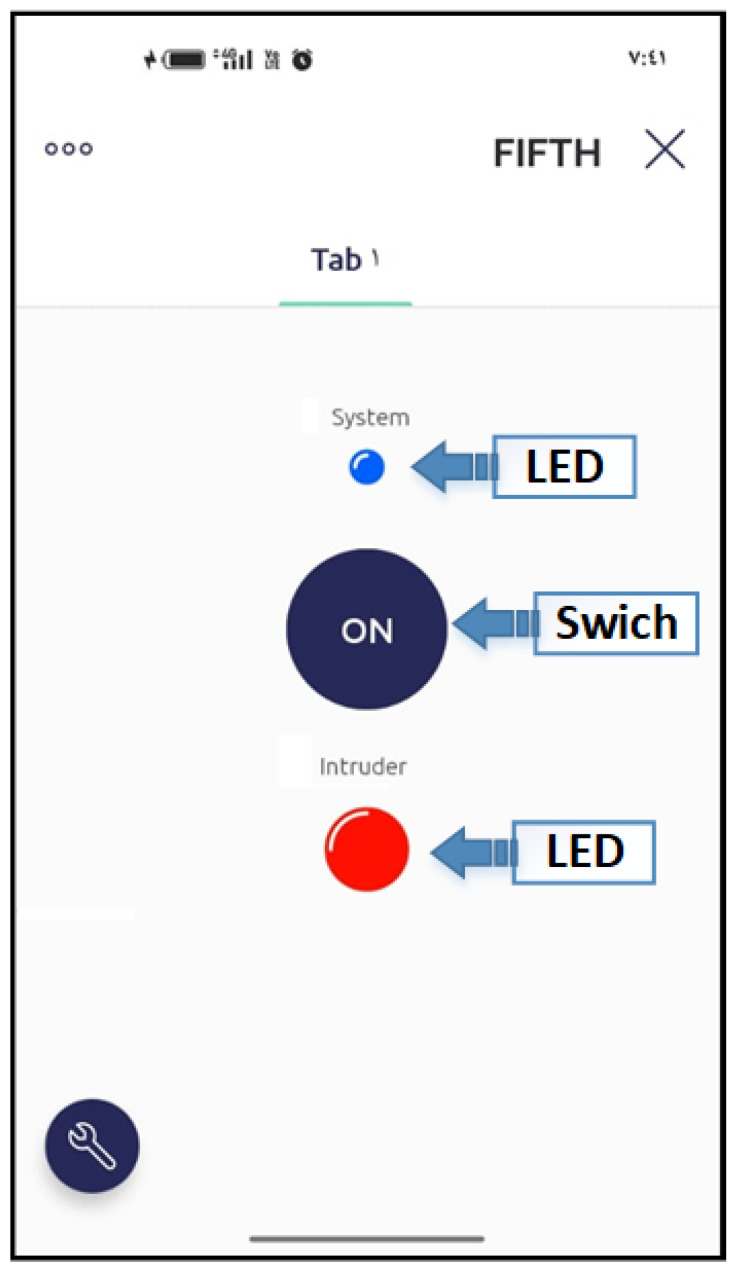
Mobile App window.

The prototype was able to detect all generated events in real time, where a notification was sent immediately (in less than 3 s) via the cloud. Figure 13 shows a control panel of the proposed detection system on the mobile. This app is built using Blynk app builder, which uses drag and drop pre-designed elements to build a custom user interface (UI) [20]. It can be seen that there are two light-emitting diode (LED) indicators (System and Intruder) and one controlling switch. The LED, named the system, will be continuously flashing when there is no problem in the connection between the prototype and the mobile app through the cloud. The LED, named the intruder, is turned on when an intruder is detected by the prototype. At the beginning and once the threshold is computed, the prototype goes into idle mode, waiting for a switching signal that will come from the mobile app to start the monitoring mode, as shown in Figure 4.

## 4. Conclusions

In this paper, we successfully built a real-time intruder-detection system that is based on the adaptive STA/LTA algorithm. The algorithm was implemented using an Arduino Due microcontroller and was used to detect abnormal activities in acoustically recorded signals. Prior to realizing the system, intensive studies were conducted to select the appropriate parameters for the algorithm. These studies were conducted by developing MATLAB codes to analyze a real dataset to select the algorithm’s parameters values for better performance. These parameters include the short and long window sizes, moving step size, and the detection scaling factor η. The algorithm was then implemented using the proposed hardware and tested in a real environment. The system was able to successfully detect all abnormalities generated in the area under consideration.

It is relevant to mention here that while the proposed system enjoys the advantages of being cost-effective, privacy-preserving, and relatively reliable, its implementation on the Arduino Duo device poses certain limitations. Specifically, the Arduino Duo is of limited processing capability, which hinders the use of more advanced intruder-detection algorithms. Future studies could consider other IoT devices with higher processing capabilities. Further, it is possible to use different cost-effective sensors and fuse their results for the sake of developing more reliable intruder detection systems.

## Figures and Tables

**Table 2:** Event’s average duration.

Event	Duration (s)
Use of keys	0.219
Falling of a small object	0.1485
Shrinking a plastic bag	1.036
Speaking	0.2606
Footsteps	0.29
Light switch	0.0433
Dragging table	0.5752
Wardrobe	1.033
Open door	0.38
Close door	0.91
Average	0.49

**Table 3:** Detection performance parameters of the first study. D, M, and F represent the number of actual, missed, and falsely detected events, respectively, whereas, SE, AV, and LE represent STA window sizes with 0.043, 0.49, and 1.036 s, respectively.

	Keys			Small Object			Plastic Bag			Speaking			Steps			Switch		
	D	M	F	D	M	F	D	M	F	D	M	F	D	M	F	D	M	F
SE	10	0	8	10	0	10	10	0	9	10	0	7	10	0	6	10	0	8
AV	10	0	9	10	0	13	10	0	1	10	0	10	10	0	3	20	0	10
LE	10	0	7	10	0	6	10	0	1	10	0	6	10	0	4	10	0	5
	**Drag Table**			**Wardrobe**			**Open Door**			**Close Door**			**Background**					
	**D**	**M**	**F**	**D**	**M**	**F**	**D**	**M**	**F**	**D**	**M**	**F**	**D**	**M**	**F**			
SE	10	0	5	10	0	21	10	0	30	10	0	25	0	0	18			
AV	10	0	2	10	0	8	10	0	10	10	0	9	0	0	2			
LE	10	0	2	10	0	7	10	0	7	10	0	6	0	0	4			

**Table 4:** Detection performance parameters of the key signal.

STA	*k* = 1			*k* = 5			*k* = 10			*k* = 20		
	D	M	F	D	M	F	D	M	F	D	M	F
SE = 0.043	18	0	8	14	0	4	11	0	1	10	0	0
AV = 0.49	19	0	9	19	0	9	19	0	9	19	0	9
LE = 1.036	17	0	7	16	0	6	16	0	6	15	0	5

**Table 5:** Effect of step size on the number of false events.

STA	Step Size	*k* = 1	*k* = 5	*k* = 10	*k* = 20
SE = 0.043	14	8	4	1	0
	28	8	1	0	0
	42	8	1	0	0
	56	8	0	0	0

**Table 6:** Estimated values of η and average computation time using the developed system and MATLAB code.

Recorded	BG #1	BG #2	BG #3	BG #4	BG #5	Avg. Time (ms)
System	2.1	1.95	2.2	2.25	2.25	523.36
MATLAB	2.1	1.95	2.2	2.25	2.25	1.71

**Table 7:** Number of detected events in the recorded data using the developed system and MATLAB code.

Record	D #1	D #2	D #3	D #4	D #5
System	5	5	5	5	5
MATLAB	5	5	5	5	5

## Data Availability

Not applicable.
